# Age, period and cohort effects on adult physical activity levels from 1991 to 2011 in China

**DOI:** 10.1186/s12966-016-0364-z

**Published:** 2016-04-20

**Authors:** Jiajie Zang, Shu Wen Ng

**Affiliations:** Department of Nutrition Hygiene, Division of Health Risk Factor Monitoring and Control, Shanghai Municipal Center for Disease Control and Prevention, Shanghai, China; Department of Nutrition, Gillings School of Global Public Health, University of North Carolina at Chapel Hill, Chapel Hill, NC USA; Carolina Population Center and Gillings School of Global Public Health, University of North Carolina, 137 E. Franklin St., CB # 8120, Chapel Hill, NC 27516 USA

**Keywords:** Physical activity, Age, Period, Cohort, Adults, Environment, China

## Abstract

**Background:**

To date no work has differentiated the effects of age, period, and cohort on physical activity (PA) among Chinese adults, while also considering biological, behavioral, economic, and environmental factors over time.

**Methods:**

We used data from the China Health and Nutrition Survey (CHNS) between 1991 and 2011 (20 years). The outcomes of interest are metabolic equivalent of task (MET) hours per week from work and domestic activities. Age, individual characteristics, household size, asset ownership, urbanization were included as covariates. Analyses for adult (≥20y) males (*n* = 29,343) and females (*n* = 31,094) was conducted to explicitly assess differences in PA due to age vs period effects, and implicitly assess differences by cohorts due to the period-specific experiences across individuals of varying ages.

**Results:**

The mean age of the sample rose from 41.31 to 50.8 years and PA decreased from 427.75 ± 264.35 MET hours per week (MET-hr/wk) in 1991 to 245.99 ± 206.65 MET-hr/wk in 2011, with much steeper declines for women compared to men. For both genders, we found non-linear decreases in PA with age over time. Controlling for age effects, negative period effects on PA were observed in each survey year, and were substantial from 1993 to 2000 for males and from 1993 to 2011 for females. The interaction between survey year and age (*P* < 0.05) were observed from 2004 to 2011. Higher community urbanicity, vehicle ownership, TV and computer ownership, overweight and obese, higher education served as negative predictors. Bicycle ownership, bigger household size, non-professional jobs, being married and having more children (for women) were positive predictors of PA (*P* < 0.05). Furthermore, at any given age, individuals who were younger at baseline had higher mean PA compared with individuals older at baseline.

**Conclusion:**

This study followed a large cohort of adults over a significant portion of their lives. Strong age and secular trends were observed, resulting in an increasing number of participants who have or are likely to lower their PA levels. These trends suggest that tackling the rapid PA decline among its population is of high priority for China’s public health outlook as its population ages and continues to experience significant economic and environmental changes.

## Background

Over the last few decades, China has experienced reductions in mortality and morbidity attributable to infectious diseases, but concurrently has had rapid increases in the prevalence of chronic non-communicable diseases (NCDs) [1]. Alongside this epidemiological transition is China’s rapidly aging population. Consequently, the burden of NCDs in China has dramatically increased and contributes to a significant and growing proportion of deaths as well as rising health care costs in recent years [2, 3].

There is now clear consensus that physical activity (PA) helps reduce the risk of NCDs such as cardiovascular and cerebrovascular diseases, diabetes, obesity, hypertension, cognitive function and cancers [4, 5], and also has positive effects on many aspects of mental health [6]. However, while the benefits of PA are well accepted, unprecedented social and economic changes in China have led to dramatically lower levels of PA at work, as jobs have become less physically demanding over time. Moreover, economic and technological improvements have facilitated house work and transportation, increasing sedentary time across the country in recent years [7]. These changes have been found to be strongly associated with large increases in the prevalence of overweight and obesity across all ages, regions, and levels of socioeconomic status [8, 9], as well as in the rise of NCDs [5] in China.

Indeed, engagement in various types or forms of PA is likely related to age, life-course, socioeconomic factors, assets ownership, the built environment and urbanization. Therefore, measurements of these factors are important for understanding how changes in PA may alter risk for disease or mortality. For a country like China that has an aging population, has and continues to experience rapid economic and environmental changes, understanding how these factors relate to PA is particularly important. However, most PA data available in China are cross-sectional [6, 10–12], so past studies using such data to study the associations between exposures and PA between age groups may represent differences between birth cohorts rather than effects of exposures [6, 10–12]. In contrast, studies using longitudinal designs may reflect temporal (period) factors. Thus, it is difficult to distinguish among effects of aging, cohorts, and period when either a cross-sectional or a longitudinal design is used.

To address these gaps, this study describes changes in PA among Chinese adults using 20-years of longitudinal data. We explicitly assess differences in PA within individuals over time (age effect) and population-wide differences in PA overtime (period effect), and implicitly assess differences in the experienced period effect across individuals of varying ages (cohort effect). We do so while also adjusting for social, economic, and environmental factors over time to determine how these factors contribute to changes in PA levels.

## Methods

### Data

We used data from the China Health and Nutrition Survey (CHNS), a prospective household-based study that includes multiple ages and cohorts across nine rounds of surveys between 1989 and 2011 in nine diverse provinces and three megacities (Beijing, Shanghai and Chongqing were added in 2011) [13]. A multistage, stratified sampling design was used to ensure that the CHNS provided representation of rural, urban and suburban areas varying substantially in geography, economic development, public resources and health indicators [13]. The CHNS is the only large scale, longitudinal study of its kind in China. Further information on survey procedures and rationale of the CHNS is in the cohort profile [13]. The CHNS was approved by the institutional review committees of the University of North Carolina at Chapel Hill, the National Institute of Nutrition and Food Safety and China Center for Disease Control and Prevention.

Our analysis included the eight rounds of survey data collected in 1991, 1993, 1997, 2000, 2004, 2006, 2009 and 2011. We did not use data from 1989 (*n* = 4753) because the sample at that wave only included adults 20–45 years old. We excluded participants who had fewer than two waves of data collection (including participants from the three mega-cities), as we would be unable to assess temporal trends for these individuals (*n* = 10,527). Additionally, observations were excluded from the analysis if a participant was pregnant, disabled or retired at the time of data collection. We focused our analyses on adults who were 20–59y in 1991. We omitted adults >60y in 1991 from our analysis due to the small and shrinking sample size over time as this cohort aged and some respondents passed away. Consequently, the inclusion of the >60y birth cohort (that would have been >80y by 2011) would have introduced potential bias.

Our final analysis sample included 13,245 participants, with an average of 4.5 observations collected on each subject (60,437 observations). The data are an unbalanced panel, with some individuals being absent in certain waves and returning in future waves as the sample was replenished over time [13, 14]. If a community had <20 households recruited, the CHNS team randomly selected additional households to obtain at least 20 households in each community. Individuals who were members of ‘old’ (previously sampled) and ‘new’ households helped replenish the sample. Relative to previous rounds of data collection, retention was between 80 and 88 % across all surveys after 1991 [13].

### Outcomes

The outcomes of interest are the metabolic equivalent of task (MET) hours per week (MET-hr/wk). MET is defined as the ratio of a person’s working metabolic rate relative to his or her resting (basal) metabolic rate [15]. We derived PA measures by multiplying (sub)activity-specific MET values obtained from the literature [13, 16] by the self-reported time reported spent in these (sub)activities among individuals in the CHNS to obtain the MET-hrs/week measurement. The MET-hr/wk measurement accounts for both the average intensity of each (sub)activity, and the time spent in each activity. In the CHNS, questions about occupational and domestic activities started in 1991, but questions about active leisure (from various forms of sports and exercise such as walking/strolling, jogging/running, swimming, badminton, table tennis, basketball, dance, wushu), and travel (from walking, bicycling, taking public transport, and driving) were only included starting in 1997. Measurements of sedentary leisure time among adults were only available starting in 2004. Due to our interest in measuring the changes in PA across periods, the primary outcome used is limited to work MET-hrs/wk from both market-based and home-based work, plus domestic MET-hrs/wk from preparing food, buying food, laundry and childcare (which may omit other important domestic activities such as cleaning). We term this “work and domestic PA”. Details on how these values were calculated are described elsewhere [17, 18]. To provide context, we also include descriptive findings on the amount of active leisure PA and travel PA in MET-hrs/wk and sedentary leisure time(hrs/wk) derived from self-reported frequency and time spent on weekends vs weekdays in: various forms of sports and exercise (such as walking/strolling, jogging/running, swimming, badminton, table tennis, basketball, dance, wushu); on walking, bicycling, taking public transport, and driving, and; on watching television/movies/videos, playing video/computer games and reading/writing, respectively.

### Covariates

#### Age

Age at the baseline survey was used to define age groups for analysis (20–29y, 30–39y, 40–49y, 50–59y). Age and age-squared were included in longitudinal models to account for the nonlinear association of PA with age.

#### Individual factors

We included a number of individual level measures that may be associated with PA levels, including: weight status (Normal weight/Overweight/Obese, defined using BMI of 25–30 as overweight and BMI >30 as obese), occupation, marriage status, education, and number of children.

#### Household socio-economic factors

We also accounted for a number of household factors such as household size (number of persons reported to be living in the household at the time of the survey), and household income.

#### Assets ownership

We were interested if ownership of certain technologies relate to PA and so included measurements of bicycle ownership, vehicle ownership, and TV and computer ownership.

#### Urbanization

A multi-component urbanicity index created from community survey data [19, 20] reflected population size and density, community infrastructure, and economic and environment characteristics. An increase in the value of the index over time represents greater urbanization.

### Statistical analysis

Cross-sectional univariate descriptive statistics of the primary outcomes and explanatory variables were calculated and reported as mean ± SD or %, stratified by gender (due to potential differences related to gender roles) for each survey year. For context, we also show the estimated MET-hrs/wk from active leisure and travel activities, as well as sedentary leisure time (in hrs/wk).

As the CHNS is a longitudinal study, attrition, migration, modifications to the sampling methods (including replenishment) over time, and other factors may result in cohort membership varying over time. In order to limit potential bias as a result of possible sample selection issues, we used a Heckman-two-step approach [21] to calculate an “inverse Mill’s ratio” for inclusion as a time-varying variable in all the models described below. This “inverse Mill’s ratio” is the inverse of the predicted probability that an individual was included in each survey given his/her province, community urbanicity and their interaction.

A series of longitudinal mixed effects models with fixed and random individual-level effects and random slopes were used to explicitly assess differences in PA within individuals over time (age effect), population-wide differences in PA overtime (period effect), and implicitly assess differences in the experienced period effect across individuals of varying ages (cohort effect). Mixed effects models were chosen because they can accommodate unbalanced data and continuous covariates, and because the hierarchical nature of the models overcome the identifiability problem by not assuming that age-period-cohort (APC) effects are linear and additive at the same level of analysis [22, 23].

We conducted four models. Model 1 included age and age-squared, indicator variables representing survey year, and age and age-squared interacted with survey year. The year coefficients describe the period effect, and interacted terms describe how period effects may differ by age (cohort effect). Model 2 layers on individual level covariates (occupation, weight status, marital status, education and, including number of kids for female to model 1. In addition to providing information about how these variables relate to PA, comparing model 2 to model 1 tells us whether these individual factors explain some of the period effects. Model 3 layers on household socio-economic measures such as household income, household size, household ownership and urbanization index to model 1. Similarly, comparing model 3 to model 1 tells us whether these household factors explain some of the period effects. Model 4 includes the full set of variables.

We also estimated a set of models stratified by baseline age group (which represent cohorts), and the comparison of coefficients across baseline age-stratified models provide insights into period-specific effects of covariates. Age and age-squared were omitted from these stratified models. All models were conducted using Stata’s XTMIXED program [24]. Results were considered significant at *p* < 0.05.

## Results

Cross-sectional analysis across survey years (Table 1) indicated that the mean age of the sample increased from 41.31 to 50.8 years. Over the 20-year period, mean work and domestic PA levels among Chinese adults fell by more than half, from 427.75 ± 264.35 MET-hrs/wk in 1991 to 245.99 ± 206.65 MET-hrs/wk in 2011. Work and domestic PA levels among females fell much more sharply than males (458.75 to 232.43 MET-hrs/wk vs 396.42 to 261.34 MET-hrs/wk, *p*-value for difference = 0.003). For context, we see that active leisure and travel PA contribute a small amount to PA, and did not change meaningfully between 1997 and 2011. Likewise, sedentary leisure time did not change meaningfully between 2004 and 2011, but throughout this period, males spent more time in sedentary leisure activities than females.

**Table 1:** Cross-sectional univariate descriptives of the China Health and Nutrition Survey across survey years, mean ± SD

	Survey year							
	1991	1993	1997	2000	2004	2006	2009	2011
All Participants (N)	7993	8207	7980	8548	7199	7018	7078	6414
Age (years)	41.31 ± 14.29	42.54 ± 14.65	42.11 ± 13.78	43.34 ± 13.36	46.37 ± 13.23	47.62 ± 13.03	48.89 ± 13.06	50.80 ± 12.92
Male (%)	49.7	50.2	50.1	49.7	47.1	46.2	47.5	46.9
Overweight/Obese (%)	12.2	13.9	17.2	22.2	25.0	26.7	29.1	32.9
Work & domestic MET-hours/week	427.75 ± 264.35	354.43 ± 222.28	356.38 ± 231.15	304.74 ± 210.46	263.08 ± 227.32	262.23 ± 232.78	249.46 ± 215.88	245.99 ± 206.65
Active leisure and travel MET-hours/week	Not measured		11.00 ± 15.35	12.51 ± 16.30	9.69 ± 18.43	9.28 ± 16.97	9.88 ± 17.70	11.25 ± 21.88
Sedentary leisure time (hours/week)	Not measured				18.47 ± 15.87	18.13 ± 15.06	19.57 ± 15.77	18.64 ± 15.98
Male (N)	3976	4117	4001	4250	3389	3241	3360	3009
Age (years)	40.68 ± 13.94	41.77 ± 14.26	40.90 ± 13.06	42.27 ± 12.74	45.69 ± 12.75	46.98 ± 12.43	48.30 ± 12.73	50.29 ± 12.70
Overweight/Obese (%)	9.4	11.3	15.4	21.0	23.7	26.4	29.0	32.8
Work & domestic MET-hours/week	396.42 ± 244.45	333.59 ± 208.01	353.81 ± 221.81	304.07 ± 200.34	284.95 ± 231.70	281.37 ± 236.20	257.41 ± 210.57	261.34 ± 206.88
Active leisure and travel MET-hours/week	Not measured		12.49 ± 17.15	13.89 ± 18.45	10.52 ± 21.43	10.26 ± 19.32	10.23 ± 19.30	11.94 ± 23.25
Sedentary leisure time (hours/week)	Not measured				20.38 ± 16.43	20.25 ± 16.75	21.17 ± 16.84	19.21 ± 20.05
Female (N)	4017	4090	3979	4298	3810	3777	3718	3405
Age (years)	41.94 ± 14.60	43.31 ± 14.98	43.33 ± 14.36	44.40 ± 13.86	46.98 ± 13.61	48.18 ± 13.50	49.42 ± 13.33	51.24 ± 13.10
Overweight/Obese (%)	14.8	16.2	19.0	23.2	26.2	26.9	29.3	32.9
Work & domestic MET-hours/week	458.75 ± 279.26	375.42 ± 233.93	358.96 ± 240.17	305.40 ± 220.03	243.63 ± 221.58	245.80 ± 228.55	242.28 ± 220.35	232.43 ± 205.53
Active leisure and travel MET-hours/week	Not measured		9.50 ± 13.14	11.24 ± 13.94	8.67 ± 13.82	8.12 ± 13.57	9.45 ± 15.50	10.43 ± 20.09
Sedentary leisure time (hours/week)	Not measured				17.47 ± 15.44	17.15 ± 14.12	18.60 ± 14.96	17.56 ± 14.65

Clearly, work and domestic PA remained key contributors of PA among Chinese adults. Meanwhile, the prevalence of overweight and obese in Chinese adults nearly tripled between 1991 and 2001 from 12.2 to 32.9 %.

### Adult men

**Fig. 1:**
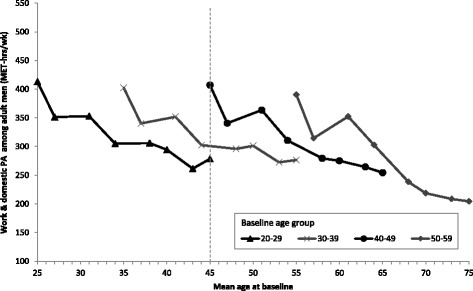
Age trends in mean work and domestic PA among adult men in the CHNS by baseline age groups (MET-hrs/wk). Notes: Points represent mean work & domestic PA level of men at the mean age for the age group in each survey year (1991, 1993, 1997, 2000, 2004, 2006, 2009, 2011). The vertical dotted line illustrates the difference in estimated PA level for a 45-year old male in 1991 vs 45-year old male in 2001 vs a 45-year old male in 2011 (cohort effect)

In Fig. 1, the average work and domestic PA levels of men in the four baseline age groups are shown according to the mean age of the group in each survey year. Each point represents a survey year, and the lines span the 20 year period over which work and domestic PA data was collected. Younger men had higher initial PA, but with age, PA levels declined across all baseline age groups. However, the relationship between age and mean work and domestic PA was non-linear. Meanwhile, the vertical dotted line illustrates the difference in estimated work and domestic PA level for a 45-year old male in 1991 (407 MET-hr/wk) compared to a 45-year old male in 2001 (300 MET-hr/wk), and a 45-year old male in 2011 (278 MET-hr/wk). This is the cohort effect.

The non-linear age-related patterns of reductions in work and domestic PA were confirmed by the longitudinal model results. Controlling for the age, negative period effects on work and domestic PA were observed in each survey year. The negative period effects on work and domestic PA were particularly substantial from 1993 to 2000, and again in 2009 and 2011 (Table 2, Model 1). The interaction between survey year and age (*p* < 0.05) were observed from 1997 to2000 (Table 2, Model 1).

**Table 2:** Mixed effects estimates on work and domestic PA levels (MET-hrs/wk) among Chinese adult men, coefficient (95 % CI)

Men	Model 1	Model 2	Model 3	Model 4
Age	**7.99 (6.65,9.33)**	**8.66 (7.43,9.9)**	**7.67 (6.39,8.95)**	**8.32 (7.02,9.61)**
Age- squared	**−0.10 (−0.12,-0.09)**	**−0.11 (−0.12,-0.09)**	**−0.09 (−0.11,-0.08)**	**−0.10 (−0.11,-0.08)**
Survey year (ref: 1991)				
1993	**−137.22 (−160.59,−113.86)**	**−110.57 (−135.29,−85.85)**	**−97.37 (−122.23,−72.50)**	**−84.94 (−110.60,−59.28)**
1997	**−163.83 (−190.22,−137.43)**	**−128.64 (−156.41,−100.86)**	**−83.24 (−111.03,−55.44)**	**−92.15 (−121.13,−63.17)**
2000	**−208.57 (−235.59,−181.56)**	**−147.80 (−176.52,−119.09)**	**−94.38 (−122.67,−66.09)**	**−91.75 (−121.94,−61.56)**
2004	**−179.27 (−211.60,−146.94)**	**−96.50 (−129.25,−63.75)**	**−47.66 (−81.60,−13.73)**	−28.36 (−63.38,6.67)
2006	**−164.98 (−201.06,−128.91)**	**−76.11 (−111.48,−40.74)**	−14.30 (−52.31,23.70)	−6.40 (−44.14,31.35)
2009	**−226.34 (−261.10,−191.59)**	**−157.02 (−189.40,−124.65)**	**−73.00 (−108.66,−37.33)**	**−98.68 (−133.43,−63.93)**
2011	**−213.19 (−251.38,−175.00)**	**−113.61 (−148.50,−78.73)**	−28.35 (−67.44,10.74)	**−41.33 (−78.83,−3.83)**
1993*Age	0.1 0 (−0.42,0.62)	0.01 (−0.53,0.55)	−0.09 (−0.65,0.47)	−0.27 (−0.84,0.30)
1997*Age	**1.06 (0.46,1.66)**	**0.75 (0.13,1.37)**	0.55 (−0.08,1.18)	0.36 (−0.30,1.01)
2000*Age	**0.86 (0.26,1.47)**	0.18 (−0.45,0.81)	0.40 (−0.23,1.03)	−0.35 (−1.02,0.31)
2004*Age	−0.43 (−1.12,0.26)	**−0.74 (−1.44,−0.03)**	**−0.77 (−1.49,−0.06)**	**−1.39 (−2.14,−0.64)**
2006*Age	−0.65 (−1.40,0.10)	**−1.20 (−1.94,−0.46)**	**−1.00 (−1.78,−0.22)**	**−1.74 (−2.53,−0.95)**
2009*Age	0.29 (−0.43,1.02)	0.14 (−0.55,0.82)	0.11 (−0.62,0.84)	−0.29 (−1.01,0.43)
2011*Age	0.01 (−0.76,0.78)	−0.59 (−1.30,0.13)	−0.50 (−1.28,0.27)	**−1.25 (−2.00,−0.49)**
Inverse Mill’s Ratio	**−6.3 (−6.95,−5.66)**	**−4.44 (−5.06,−3.82)**	**−4.56 (−5.21,−3.90)**	**−3.62 (−4.25,−3.00)**
Household income			0.00 (0.00,0.00)	0.00 (0.00,0.00)
Bicycle ownership			**6.14 (0.23,12.04)**	**10.29 (4.56,16.03)**
Vehicle ownership			**−13.06 (−19.00,−7.11)**	**−6.53 (−12.43,−0.64)**
TV and computer ownership			**−39.67 (−48.30,−31.04)**	**−14.43 (−23.12,−5.74)**
Household size			**3.23 (1.44,5.02)**	**−1.85 (−3.60,−0.11)**
Community Urbanicity			**−5.01 (−5.19,−4.83)**	**−1.80 (−1.99,−1.60)**
Overweight/Obese		**−11.61 (−17.95,−5.27)**		**−10.08 (−16.6,−3.56)**
Occupation (ref: professional job)				
Farmer		**322.89 (314.63,331.15)**		**305.44 (296.18,314.70)**
officer/soldier/athlete		**54.74 (27.66,81.83)**		**64.05 (35.92,92.19)**
Unskilled worker		**188.91 (179.61,198.21)**		**185.47 (175.93,195.00)**
Skilled worker		**84.52 (75.66,93.38)**		**85.98 (76.99,94.97)**
Other occupation		**49.06 (36.36,61.77)**		**48.01 (34.47,61.55)**
Married		**7.40 (2.53,12.28)**		**5.69 (0.61,10.76)**
Education (ref: < primary school)				
Middle and high school		**−7.25 (−13.72,−0.78)**		−2.35 (−8.97,4.28)
College and above		**−27.82 (−41.23,−14.41)**		−8.94 (−22.78,4.9)
Number of observations	28002	21675	25262	20039
Number of individuals	7042	6601	6923	6456

Notes: Data from CHNS 1991–2011; bold values denote statistical significance at *p* < 0.05

Model 1 included age and age-squared, indicator variables representing survey year, and age and age-squared interacted with survey year

Model 2 layers on individual level covariates (occupation, weight status, marital status, education to model 1

Model 3 layers on household socio-economic measures such as household income, household size, household ownership and urbanization index to model 1

Model 4 includes the full set of variables

In Model 2, we found that higher community urbanicity, vehicle ownership, TV and computer ownership were negative predictors of work and domestic PA. Meanwhile bicycle ownership and bigger household size were positive predictors of work and domestic PA (Table 2, Model 2). Given that these measurements of technologies that should not directly affect work or domestic PA are associated suggests that there is strong correlation across various domains of PA.

In Model 3, we found that being overweight or obese was negatively related to PA. Compared to professional jobs (e.g., doctor, professor, lawyer, architect, engineer, editor), other occupations (farmer, officer/soldier/athlete, unskilled worker, skilled worker, students) were positive predictors of work and domestic PA. Being married was positively associated, but higher education was negatively associated with work and domestic PA levels (Table 2, Model 3). In comparing the coefficients from Model 3 to Model 2, we see that household factors account for much of the period effects.

Indeed, after adjusting for potential confounding effects of household socio-economic factors and urbanicity, as well as individual measures (Table 2, Model 4), the direction of all of the period effects remained the same, but coefficients were slightly attenuated relative to Model 2. Moreover, the inclusion of both household and individual factors strengthen the cohort effects (how period effects vary by age) especially for 2004*age, 2006*age and 2011*age.

**Fig. 2:**
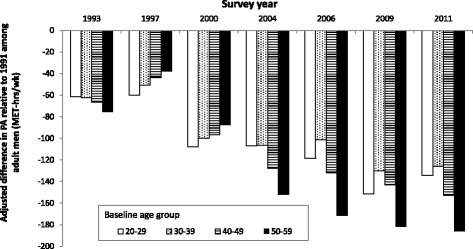
Secular trends in work and domestic PA level among adult men in CHNS by baseline age groups. Notes: Bars represent difference from baseline (1991) work & domestic PA, estimated from longitudinal models, stratified by baseline age groups

Lastly, age-stratified models show that in general, adult men who were younger at baseline (white bars) had lower decreased mean work and domestic PA levels compared with individuals older at baseline (darker bars). The predicted physical activity levels decreased from 62.9 to 146.5, 62.6 to 152.1, 67.2 to 199.2, 75.9 to 250.4 MET-hrs/wk from 1993 to 2011 for male population of baseline aged 20–29, 30–39, 40–49, and 50–59 respectively. Figure 2 presents the predicted work and domestic PA values relative to baseline (1991).

### Adult women

**Fig. 3:**
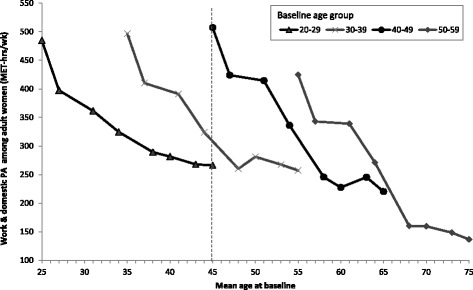
Age trends in mean work and domestic PA among adult women in the CHNS by baseline age groups (MET-hrs/wk). Notes: Points represent mean work & domestic PA level of women at the mean age for the age group in each survey year (1991, 1993, 1997, 2000, 2004, 2006, 2009, 2011). The vertical dotted line illustrates the difference in estimated PA level for a 45-year old woman in 1991 compared to a 45-year old woman in 2001, and 45-year old woman in 2011 (cohort effect)

Mean work and domestic PA of women in four age groups is shown in Fig. 3. Unlike men, women between 25 and 45y had comparable levels of work and domestic PA initially, while 55y women had lower baseline PA levels (the first point for each line). Nonetheless, work and domestic PA levels declined across all baseline age groups with age, and the decline for women is much starker than for men. Again, the relationship between age and mean work and domestic PA is non-linear. The cohort effect is also stronger among women (compared to men), as a 45-year old woman in 1991 expends 507 MET-hrs/wk compared to the 226 MET-hrs/wk a 45-year old woman in 2011 expends.

Similar to men, the non-linear age-related patterns of reductions in work and domestic PA was confirmed by the longitudinal model results among women. Controlling for age, there were large negative period effects on work and domestic PA were observed in each survey year (Table 3, Model 1) and positive cohort effects (how period effects vary by age). In general the results for women were consistent with those of men albeit with larger coefficients. Estimation results for women were also consistent with those for men in Model 2 and 3, with the exception of weight status (not statistically significant for women) (Table 3, Model 3). Again, in comparing the year coefficients from Models 2 to 3, we see that household factors account for much of the period effects (year effects are smaller in Model 2 as the household factors explain more of the PA changes).

**Table 3:** Mixed effects estimates on work and domestic PA levels (MET-hrs/wk) among Chinese adult women, coefficient (95 % CI)

Women	Model 1	Model 2	Model 3	Model 4
Age	**11.52 (10.25,12.79)**	**7.51 (6.06,8.96)**	**13.07 (11.7,14.45)**	**7.26 (5.67,8.84)**
Age- squared	**−0.16 (−0.18,−0.15)**	**−0.11 (−0.13,−0.1)**	**−0.17 (−0.18,−0.15)**	**−0.1 (−0.12,−0.08)**
Survey year (ref: 1991)				
1993	**−191.08 (−215.13,−167.03)**	**−174.74 (−201.52,−147.96)**	**−161.92 (−189.94,−133.89)**	**−154.8 (−183.4,−126.19)**
1997	**−247.85 (−274.67,−221.04)**	**−231.38 (−262.37,−200.4)**	**−190.69 (−221.93,−159.45)**	**−194.79 (−227.93,−161.64)**
2000	**−306.59 (−333.95,−279.24)**	**−241.57 (−274.56,−208.57)**	**−183.96 (−215.54,−152.38)**	**−187.61 (−222.63,−152.6)**
2004	**−356.11 (−389.18,−323.03)**	**−172.42 (−212.01,−132.83)**	**−176.80 (−215.67,−137.92)**	**−117.34 (−159.23,−75.45)**
2006	**−352.57 (−386.89,−318.26)**	**−172.04 (−211.6,−132.49)**	**−141.05 (−181.73,−100.38)**	**−112.65 (−155.36,−69.94)**
2009	**−386.64 (−420.80,−352.48)**	**−190.23 (−228.17,−152.29)**	**−186.09 (−225.02,−147.16)**	**−140.05 (−180.76,−99.33)**
2011	**−401.56 (−438.85,−364.26)**	**−184.7 (−225.63,−143.76)**	**−188.98 (−231.32,−146.64)**	**−147.06 (−190.87,−103.25)**
1993*Age	**0.57 (0.06,1.08)**	0.55 (−0.05,1.14)	**0.71 (0.07,1.34)**	0.37 (−0.28,1.03)
1997*Age	**1.36 (0.78,1.93)**	**1.88 (1.18,2.57)**	**1.61 (0.91,2.32)**	**1.41 (0.66,2.16)**
2000*Age	**1.40 (0.82,1.98)**	**1.11 (0.38,1.84)**	**0.95 (0.25,1.65)**	0.54 (−0.24,1.32)
2004*Age	**0.99 (0.32,1.66)**	−0.72 (−1.58,0.14)	−0.32 (−1.14,0.50)	**−1.29 (−2.2,−0.38)**
2006*Age	**1.04 (0.34,1.73)**	−0.57 (−1.42,0.27)	−0.48 (−1.32,0.37)	**−1.01 (−1.93,−0.1)**
2009*Age	**1.86 (1.17,2.55)**	0.08 (−0.71,0.88)	**0.83 (0.03,1.62)**	−0.37 (−1.23,0.48)
2011*Age	**1.81 (1.08,2.54)**	−0.41 (−1.24,0.43)	0.74 (−0.10,1.59)	−0.62 (−1.52,0.27)
Inverse Mill’s Ratio	**−7.76 (−8.44,−7.08)**	**−5.88 (−6.54,−5.21)**	**−5.77 (−6.48,−5.05)**	**−5.14 (−5.82,−4.46)**
Household income			0.00 (0.00,0.00)	0.00 (0.00,0.00)
Bicycle ownership			2.94 (−3.41,9.30)	2.69 (−3.74,9.11)
Vehicle ownership			**−12.32 (−18.77,−5.87)**	**−7.32 (−13.91,−0.72)**
TV and computer ownership			**−40.41 (−49.68,−31.13)**	**−20.89 (−30.17,−11.61)**
Household size			**6.77 (4.84,8.70)**	−0.73 (−2.72,1.26)
Community Urbanicity			**−5.23 (−5.42,−5.04)**	**−1.94 (−2.18,−1.71)**
Overweight/Obese		−5.28 (−12.07,1.52)		−3.04 (−10.02,3.94)
Occupation (ref: professional job)				
Farmer		**323.73 (313.07,334.39)**		**295.27 (283.14,307.39)**
officer/soldier/athlete		**84.26 (37.41,131.12)**		**80.93 (29.79,132.08)**
Unskilled worker		**205.69 (193.04,218.34)**		**199.06 (186.15,211.97)**
Skilled worker		**73.93 (62.91,84.94)**		**72.75 (61.53,83.97)**
Other occupation		**50.52 (35.01,66.02)**		**46.78 (30.14,63.42)**
Married		1.67 (−3.29,6.63)		4.56 (−0.68,9.79)
Number of children		**10.69 (8.02,13.36)**		**8.4 (5.62,11.19)**
Education (ref: < primary school)				
middle and high school		**−27.5 (−34.83,−20.17)**		**−16.55 (−24.07,−9.02)**
college and above		**−40.66 (−58.51,−22.82)**		**−21.52 (−39.73,−3.31)**
Number of observations	29564	19654	23617	18295
Number of individuals	7293	6308	6684	6054

Notes: Data from CHNS 1991–2011; bold values denote statistical significance at *p* < 0.05

Model 1 included age and age-squared, indicator variables representing survey year, and age and age-squared interacted with survey year

Model 2 layers on individual level covariates (occupation, weight status, marital status, education and including number of kids for female to model 1

Model 3 layers on household socio-economic measures such as household income, household size, household ownership and urbanization index to model 1

Model 4 includes the full set of variables

Likewise, after adjusting for potential confounding effects of household socio-economic factors and urbanicity as well as individual measures (Table 3, Model 4)., the direction of all of the period effects remained the same, but coefficients were slightly attenuated relative to Model 2. Moreover, the inclusion of both household and individual factors strengthen the negative cohort effects especially for 2004*age and 2006*age.

**Fig. 4:**
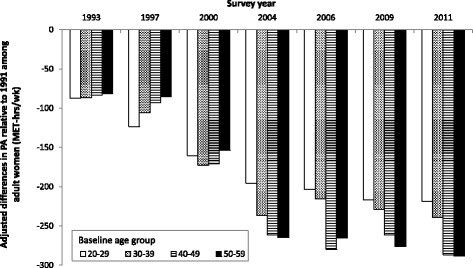
Secular trends in work and domestic PA level among adult women in CHNS by baseline age groups. Notes: Bars represent difference from baseline (1991) work & domestic PA, estimated from longitudinal models, stratified by baseline age groups

Plots of the survey year coefficients for baseline age group- and gender-stratified longitudinal mixed effects models predicting work and domestic PA further illustrate the cohort effect among adult women (Fig. 4), which is much more pronounced than among adult men.

## Discussion

The present study described changes in the primary sources of PA (work and domestic activities) among Chinese adults between 1991 and 2011, based on a questionnaire on time use that has been used in earlier studies [9, 17, 18, 25–28]. We found that throughout this 20-year period, for both adult men and women in China, work and domestic PA levels fell by nearly half, and the decline was more pronounced for women. Period effects, along with higher community urbanicity, vehicle ownership, TV and computer ownership, higher education, being overweight or obese were negatively associated with work and domestic PA levels. Bicycle ownership, labor-intensive jobs, being married, and larger household size were found to be positively associated with work and domestic PA levels. Moreover, our implicit measurement of the cohort effects by including interactions of year with age showed that being older in 2004 and 2006 had stronger negative associations with lower work and domestic PA in particular.

The CHNS was designed to provide representation of rural, urban and suburban areas with a focus on examining household- and individual-level sociodemographic factors, diet, physical activity, health and behavior changes within the context of community-level urbanicity and social and economic change [13]. While many of the measures related to health and behaviors are based on self-report, the CHNS is the only large-scale, longitudinal study of its kind in China [14]. Other PA-related studies in China, such as the cross-sectional InterASIA study conducted in 2000–2001 [10] as well as the multi-country World Health Survey studies in 2002–2003 that uses the International Physical Activity Questionnaire (IPAQ) [29] ask about participation and estimate prevalence of physical inactivity, so the measurements are not directly comparable. However, the overall findings are consistent with what we found here (occupational PA is the most prevalent).

China’s dramatic economic, demographic and social transformation continues to unfold with cohorts experiencing rapid environmental change at different ages. The changes include increased modernization of the Chinese food system; continued use and greater dissemination of modern technology in manufacturing, transportation and leisure; housing, health system and educational system changes; and new pressures on the labor, environmental and social systems [30, 31].

Since economic reform in China in the late 1980s to early 1990s, more and more workers have transitions from heavy industry to service industry jobs. Consequently, the portion of the population engaged in vigorous labor has fallen and replaced by work activities that are lighter in physical labor needs or sedentary in nature [27]. Moreover, technological advances in the workplace have lessened the physical demands of many jobs, and studies in urban China have found that these technological improvements are associated with declines in PA [18].

Concurrently, the public transportation system was improved with more buses and subways, while increase wealth have resulted in greater vehicle ownership (rose by over 15-fold between 1991 and 2011) [32], and higher reports of people using private cars as their main mode of transportation [26, 33, 34]. The proportion of Chinese who own color televisions, have access to cable networks and computers have all also risen dramatically, as have screen time [18, 35, 36]. We found consistent results in this study that these transformations are associated with the decline in PA in these two decades.

Unsurprisingly, we found that higher educational attainment was negatively associated with work and domestic physical activity. Likewise, vehicle and computer ownership, having a professional job, and living in a more urbanized area were negatively associated. Conversely, bicycle ownership, non-professional jobs, being married and bigger household size were positively associated with work and domestic PA. Certainly these factors are correlated with each other as participants who had higher education are more likely to have less labor-intensive occupations, more likely to have private cars (over bicycles) and live in urban areas (authors own review of correlation coefficients across measures). Nonetheless, the results indicate that they are also independently associated with reduced work and domestic PA levels among Chinese adults.

In this study, we found that work and domestic PA among women declined more substantially than among men. This is consistent with earlier findings [17], and largely driven by that fact that Chinese women historically do the majority of domestic activities (e.g., cooking, laundry, childcare), and still do, but both time spent and energy expended in these activities have fallen dramatically for women, while not changing much for men. This is likely due to at least two reasons. First, Chinese women have been bearing fewer children as a result of both the one-child policy (which was only recently altered), as well as for economic reasons. As seen in the models, the positive relationship between the number of children and PA suggest that the shrinking childbirth rate lessens PA from tending to children and related housework. Secondly, modern home-technologies, such as washing machines, microwaves, electric cookers, vacuum cleaners help facilitate housework by both reducing time spent as well as energy needs.

A strength of this study is that this is the largest longitudinal analysis of physical activity to be conducted in a diverse sample of Chinese adults. To our knowledge, we are the first to describe 20-year changes in PA among Chinese while differentiating effects of age, period, and birth cohort and determine how physical activity is influenced by biological, behavioral, economic, and environmental factors over time.

Nonetheless, there are several limitations to this study. Our analysis only focuses on self-reported work and domestic activity, which forms the majority of time-use as well as energy expenditure in China as well as in other developing countries [17, 37]. These measures of work and domestic activity may be misreported or incomplete (e.g., missing cleaning as a domestic activity), and partly relies on MET-values derived from the US, which may not be appropriate.

Additionally, due to data limitations (missing 2 earlier waves) we were unable to include other forms of PA in our analyses. It would be particularly important to also consider the role of leisure-time activity. Past studies have shown that in China, participation in moderate or vigorous leisure-time physical activity was low at 7.9 and 28.9 %, among urban and rural Chinese adults, respectively [10]. Other descriptive studies looking at trends over time using the CHNS also suggest that active leisure activity is increasing slightly, but insufficiently to make up the large reductions from the other domains of work, home and transportation [17]. Much more needs to be done to move the needle of active leisure, which has been found to benefit not just physical health but also mental health [6]. Investments into age and culturally appropriate efforts that use and encourage the provision of easily accessible communal spaces for tai-chi, wushu (martial arts), dance, badminton, jogging and other more popular regional or cultural sports should be made in both urban and rural settings. Greater commitments towards active transportation (e.g., bicycling via providing a bicycle-sharing system and improving road safety) should also be considered as it can concurrently address air pollution, congestion and climate change concerns. Many of these approaches are also mutually reinforcing, as improved air quality and improved traffic safety removes barriers to exercising outdoors. Indeed “whole of government” and “whole of society” approaches will be necessary [38].

The urgency of this issue is highlighted by estimates that in 2007, the direct and indirect costs of physical inactivity in China was 6.7 billion USD (in 2007 dollars) as the lack of PA contributes to between 12 and 19 % of the five major NCDs in China [23], and 8.3 % of all-cause mortality [39]. A more recent study found that cardiometabolic risk prevalence was high in all age groups in China, particularly younger adults, and that low PA consistently predicted higher cardiometabolic risk across most outcomes and age-sex groups [40]. Consequently, the economic cost of physical inactivity is expected to grow as PA levels continue to fall, given the strong age effect and secular trends found in this study, China’s rapidly aging population, as well as the much lower PA levels of the younger cohorts. This is a problem that has gained notice, and there are increasingly multi-sectoral, government policies and corporate social responsibility efforts underway to reduced inactivity, encourage PA across all domains of life, and thus to halt the growing prevalence of NCDs in China [38]. Examples include the 2007 Ministry of Health campaign “*ten thousand steps a day, the balance of eating and activity and a healthy life*”, the 2003 Guidelines for Prevention and Control of Overweight and Obesity of Chinese Adults, and the 2007 School-age Children and Teenagers Overweight and Obesity Prevention and Control Guidelines [38]. Efforts targeting children in particular are critical to encourage formation of healthier habits and lifestyles. Some intervention programs have also been shown to be effective and expanded across cities and provinces, such as the Take Ten school-based PA intervention. Originally a pilot study in Beijing in 2004, Take Ten was expanded and eventually prompted the Department of Education to institute a policy of one hour of PA in schools every day [41]. It would be useful to examine what the long-term effects of such a policy would have on this younger cohort as they age.

## Conclusion

In summary, this study followed a large cohort of adults over a significant portion of their lives. As China underwent dramatic economic transitions, we observed strong age and secular trends in work and domestic PA levels, resulting in an increasing number of men and women who have or are likely to have even lower PA levels both due to aging and the combination of period and cohort differences. These trends are highly relevant for health policy and preventive health measures in the China and other countries that are now facing similar challenges.
